# Wilson’s Disease: Expect the Unexpected

**DOI:** 10.7759/cureus.2173

**Published:** 2018-02-08

**Authors:** Talal El Imad, Hassan Al Moussawi, Fady G. Haddad, Richard Felix, Stephen M Mulrooney

**Affiliations:** 1 Department of Internal Medicine, Staten Island University Hospital, New York; 2 Department of Medicine, Staten Island University Hospital, New York; 3 Department of Gastroenterology and Hepatology, Staten Island University Hospital, New York; 4 Department of Pathology, Staten Island University Hospital, New York; 5 Department of Gastroenterology, Staten Island University Hospital, New York

**Keywords:** copper, liver, ceruloplasmin

## Abstract

A 64-year-old woman, presented with abdominal distention, jaundice and resting tremor, was found to have liver injury and abnormal liver enzymes. A computed tomography (CT) scan of the abdomen and pelvis showed abdominopelvic ascites and signs of liver cirrhosis. An extensive liver disease workup was performed and came back negative; therefore, a liver biopsy was obtained and showed evidence of cirrhosis with elevated liver copper consistent with Wilson’s disease (WD). We report a unique case of late-onset WD in which the ceruloplasmin level and 24-h urinary copper excretion were all normal.

## Introduction

Wilson’s disease (WD) is a genetic disorder affecting copper metabolism resulting in copper accumulation in many organs and tissues, reduced hepatic biliary copper excretion and reduced incorporation of copper into ceruloplasmin [1]. Affected individuals are usually between the ages of 5 and 35 years, however, there are few reports where the disease presented beyond this age. The diagnosis of WD relies primarily on the clinical and laboratory evidence of abnormal copper metabolism as well as the histological evidence of copper deposition in the liver. We report a rare case of late-onset WD presenting with cirrhosis and neurological manifestations in which the ceruloplasmin level and 24-h urinary copper levels were normal.

## Case presentation

A 64-year-old female presented to our Emergency Department with bilateral lower extremity edema and jaundice. The patient denied any fever, chills, abdominal pain, vomiting, diarrhea, hematochezia or melena. One month prior to presentation she was treated with antibiotics for a diabetic foot ulcer. Her past medical history is significant for type II diabetes mellitus, hypertension, and depression. Her current medications are metformin, glipizide, amlodipine, amitriptyline, and furosemide. She denied smoking cigarettes, alcohol or drug use. Vital signs were normal. On physical exam, the patient had icteric sclera, mild abdominal distension without focal tenderness, normal bowel sounds, and extremities revealed bilateral pitting edema. Neurological exam was significant for resting tremor and ataxic gait otherwise the rest of her exam was normal.

**Figure 1:**
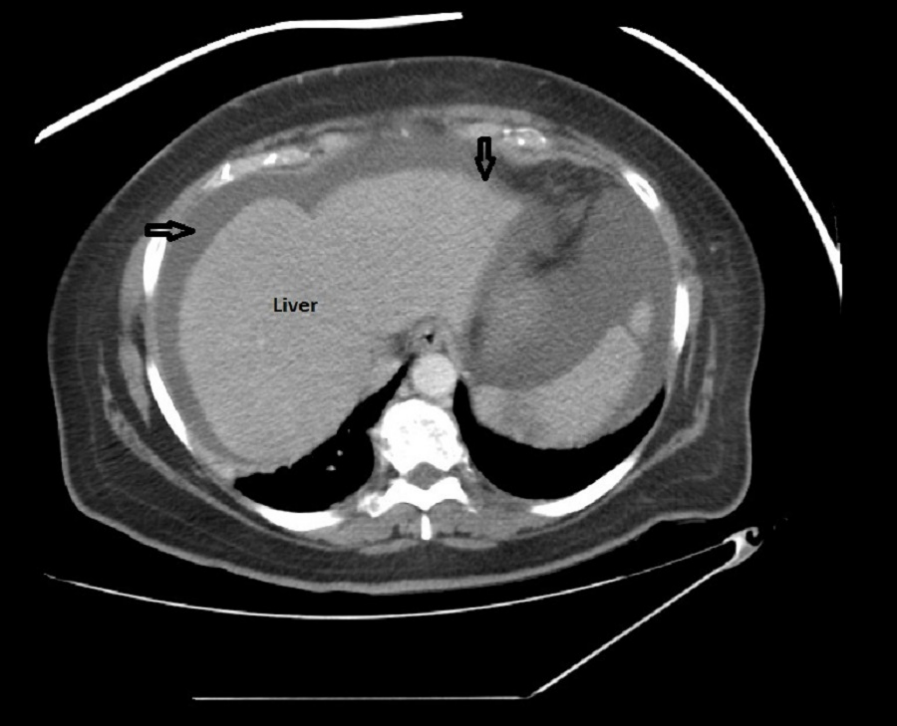
Computed tomography (CT) scan of the abdomen showing a nodular contour of the liver with moderate abdominopelvic ascites.

On presentation, laboratory studies were significant for a leukocyte count of 7000/µL, hemoglobin of 10.5 g/dl, alanine transaminase of 87 IU/L, aspartate transaminase of 224 IU/L, alkaline phosphate of 98 U/L, total bilirubin of 8.8 mg/dL, direct bilirubin of 3.2 mg/dl, albumin of 2 g/dl and international normalized ratio (INR) of 1.3. An ultrasound of the abdomen was performed and failed to show any cholelithiasis, cholecystitis or common bile duct dilation. Given her presentation and physical exam findings, a computed tomography (CT) of the abdomen with intravenous contrast injection was performed and revealed a nodular contour of the liver with mildly prominent intrahepatic biliary ducts and moderate abdominopelvic ascites (Figure 1).

**Figure 2:**
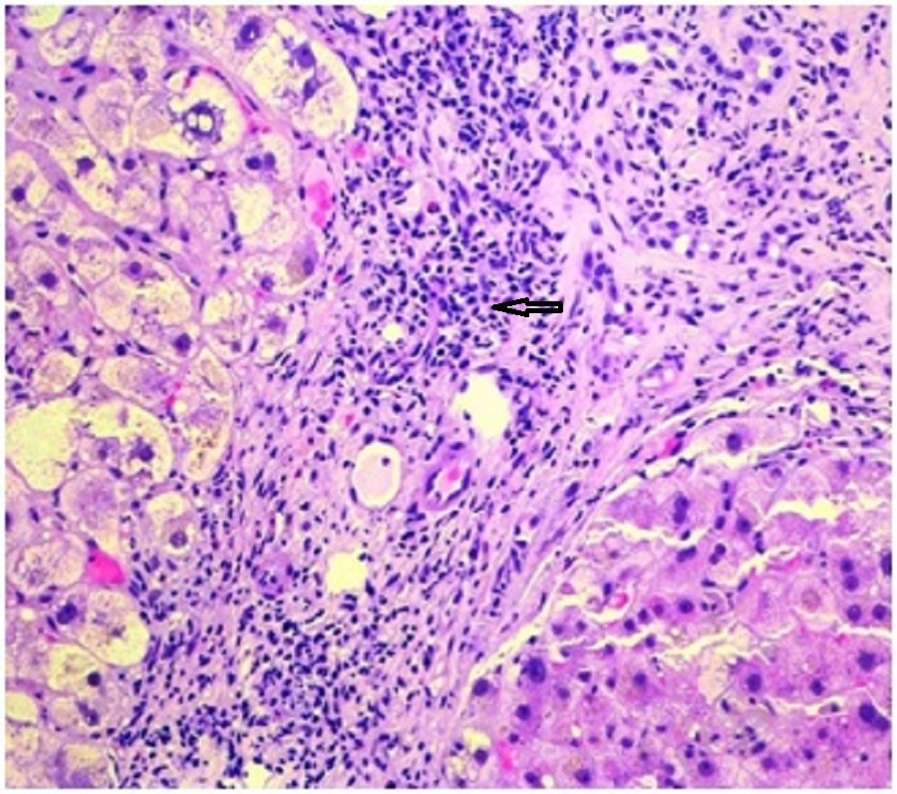
Core biopsy of the liver demonstrating portal fibrosis with chronic inflammatory infiltrate.

**Figure 3:**
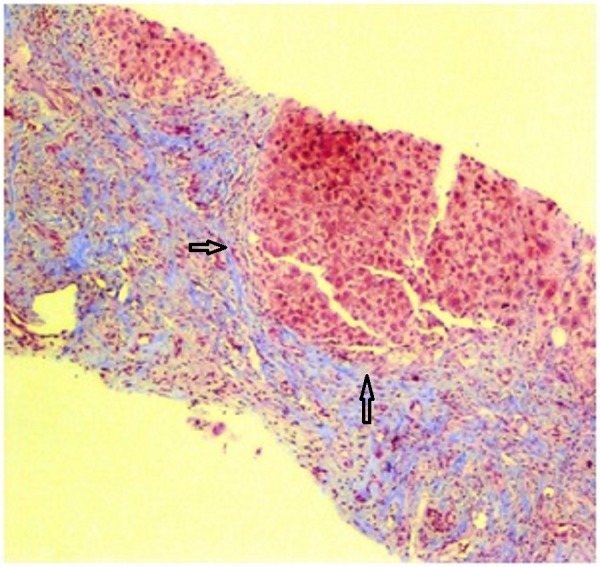
Trichrome stain highlighting the collagenous fibers surrounding nodules of hepatocytes.

A diagnostic abdominal paracentesis was performed and showed an ascitic fluid albumin of 0.5 g/dl, fluid total protein of 1.6 g/dl and therefore a calculated serum albumin ascitic fluid gradient (SAAG) of 1.5, consistent with liver cirrhosis. Ascitic fluid results were as follows: white cell count of 198 per ml with 14% segmented neutrophils, 16% lymphocytes and 70% mononuclear cells, lactate dehydrogenase (LDH) of 62 U/L, and negative cytology, gram stain and culture. Hepatitis serology, anti-smooth muscle antibodies, anti-mitochondrial antibodies were all negative. Further workup was noticeable for a serum ceruloplasmin level of 45 mg/dl, urinary copper level of 39 mcg per 24 h and transferrin saturation of 22%. An ultrasound-guided liver biopsy was performed and showed cirrhosis with hepatocytic degeneration within most of the cirrhotic nodules, and hypocellular fibrous septa surrounding the nodules (Figures 2, 3).

**Figure 4:**
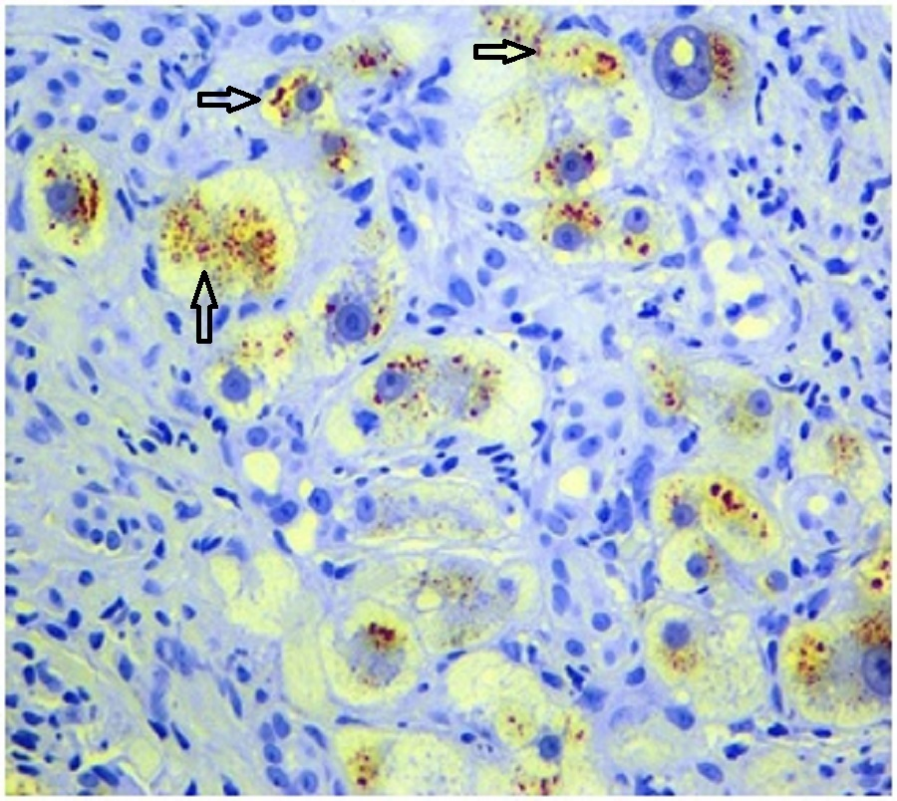
Rhodanine stain revealing the accumulation of copper within the hepatocytes.

Rhodanine stains showed prominent copper deposits in most hepatocytes (Figure 4).

A quantitative liver copper measurement confirmed the diagnosis of WD.

## Discussion

WD, an autosomal recessive disorder, is caused by a defect in copper metabolism and results in its deposition in different organs. ATP7B is a P-type ATPase expressed mainly in the liver and is responsible for the transmembrane transport of copper. Mutations in ATP7B affect the incorporation of copper into apoceruloplamin but more importantly block the excretion of copper into bile which explains the elevated levels of copper seen in this disease. WD has a wide spectrum of clinical presentations of which liver and neurological-related signs and symptoms are the most common. This inherited disorder can appear at any age, but most patients present at an age of less than 40 years. The hallmark of the diagnosis of WD is the combination of the clinical picture, the presence of Kayser–Fleischer (KF) ring on slit lamp, laboratory findings of low ceruloplasmin, high urinary copper, and high hepatic copper concentration [2]. Due to the wide range of clinical presentation and laboratory findings especially in liver-limited disease, the diagnosis of WD may become challenging. Consequently, WD is commonly underdiagnosed. Patients with WD confined to the liver may be either asymptomatic or present with the picture of decompensated liver cirrhosis, as in the case of our patient, or with acute liver failure. Our patient had no evidence of KF rings on slit lamp examination. KF ring is a common finding in patients with WD presenting with neurological symptoms, with an incidence of 90% [3]; it is absent in up to 50% of those presenting with liver disease only [4]. Interestingly, our patient had a normal, not decreased ceruloplasmin level. This may be attributable to ceruloplasmin elevation as an acute phase reaction in response to inflammation. Steindl, et al. reported that in up to 45% of liver-limited WD, serum ceruloplasmin may be normal. Therefore, the diagnosis of WD can be overlooked if one relies solely on the laboratory criteria.

Though our patient was 64 years old, WD was still in the differential after excluding other possible causes. Viral, alcoholic, ischemic, autoimmune hepatitis and hemochromatosis were each excluded in this case. Anti-mitochondrial antibodies (AMA) were negative and there was no evidence of cholestasis. Liver biopsy was done and was compatible with cirrhosis. In addition, hepatocytes were rhodanine stain positive. Confirmatory quantitative hepatic copper measurement showed elevated hepatic copper concentration. A hepatic copper concentration more than 250 mcg/g is considered diagnostic for WD especially when other possible causes of non-Wilson related liver copper deposition has been excluded [5,6]. Primary biliary cholangitis (PBC) and primary sclerosing cholangitis (PSC) could be entertained in the differential of this case, however, there were no biopsy or serological findings (AMA) consistent with PBC and neither imaging nor pathology was consistent with PSC.

## Conclusions

WD should be considered in the differential of any patient presenting with both hepatic and neurologic involvement. While typically the diagnosis of WD is based on the presence of low ceruloplasmin levels, KF rings, and elevated urinary copper excretion, this case demonstrates that these guideposts can be normal in some patients. This underscores the importance of obtaining a liver biopsy in the setting of a high clinical suspicion of the disease. This case demonstrates that in patients with hepatic and neurological findings, WD should remain a diagnostic possibility, even in older patients or in the absence of typical biochemical markers and ophthalmological findings.
